# Baseline antibody profiles predict toxicity in melanoma patients treated with immune checkpoint inhibitors

**DOI:** 10.1186/s12967-018-1452-4

**Published:** 2018-04-02

**Authors:** Michael F. Gowen, Keith M. Giles, Danny Simpson, Jeremy Tchack, Hua Zhou, Una Moran, Zarmeena Dawood, Anna C. Pavlick, Shaohui Hu, Melissa A. Wilson, Hua Zhong, Michelle Krogsgaard, Tomas Kirchhoff, Iman Osman

**Affiliations:** 1 The Ronald O. Perelman Department of Dermatology, New York University School of Medicine, New York, NY USA; 2 Applied Bioinformatics Core, New York University School of Medicine, New York, NY USA; 3 Division of Hematology & Oncology, Perlmutter Cancer Center, New York University School of Medicine, New York, NY USA; 4 Division of Epidemiology, New York University School of Medicine, New York, NY USA; 5 Department of Population Health, New York University School of Medicine, New York, NY USA; 6 Department of Pathology, New York University School of Medicine, 522 First Ave., Smilow Research Building 1311, New York, NY 10016 USA; 7 CDI Laboratories, Baltimore, MD USA; 8 Department of Medicine, New York University School of Medicine, 522 First Ave., Smilow Research Building 405, New York, NY 10016 USA

**Keywords:** Melanoma, Immunotherapy, Antibodies, Toxicity, Biomarker

## Abstract

**Background:**

Immune checkpoint inhibitors (anti-CTLA-4, anti-PD-1, or the combination) enhance anti-tumor immune responses, yielding durable clinical benefit in several cancer types, including melanoma. However, a subset of patients experience immune-related adverse events (irAEs), which can be severe and result in treatment termination. To date, no biomarker exists that can predict development of irAEs.

**Methods:**

We hypothesized that pre-treatment antibody profiles identify a subset of patients who possess a sub-clinical autoimmune phenotype that predisposes them to develop severe irAEs following immune system disinhibition. Using a HuProt human proteome array, we profiled baseline antibody levels in sera from melanoma patients treated with anti-CTLA-4, anti-PD-1, or the combination, and used support vector machine models to identify pre-treatment antibody signatures that predict irAE development.

**Results:**

We identified distinct pre-treatment serum antibody profiles associated with severe irAEs for each therapy group. Support vector machine classifier models identified antibody signatures that could effectively discriminate between toxicity groups with > 90% accuracy, sensitivity, and specificity. Pathway analyses revealed significant enrichment of antibody targets associated with immunity/autoimmunity, including TNFα signaling, toll-like receptor signaling and microRNA biogenesis.

**Conclusions:**

Our results provide the first evidence supporting a predisposition to develop severe irAEs upon immune system disinhibition, which requires further independent validation in a clinical trial setting.

**Electronic supplementary material:**

The online version of this article (10.1186/s12967-018-1452-4) contains supplementary material, which is available to authorized users.

## Background

Immune checkpoint inhibitors (ICI) target cytotoxic T lymphocyte-associated antigen 4 (CTLA-4, e.g. ipilimumab) or programmed cell death protein 1 (PD-1, e.g. nivolumab, pembrolizumab) to promote T cell mediated anti-tumor immunity and produce durable clinical benefit in a subset of patients with advanced melanoma [1]. More recently, the combination of anti-CTLA-4 and anti-PD-1 has been shown to be more efficacious than single agent therapy [2]. Despite this progress a substantial proportion of patients receiving ICI develop immune-related adverse events (irAEs) [3], which are often more severe in patients receiving combination regimens [4]. IrAEs can necessitate systemic immunosuppression therapy and/or treatment termination [5]. Hence, there is an urgent clinical need to identify patients who are more likely to develop severe irAEs, particularly as more patients receive these immune therapies due to the approval of ICI for other cancer types (e.g. bladder, lung), and in the adjuvant setting for stage III/IV melanoma [6, 7]. A biomarker predictive of immunotherapy toxicity would facilitate a personalized approach to patient management, enabling more-effective combination treatments to be used in patients who are less likely to develop severe irAEs. Additionally, identifying toxicity-prone patients would improve the clinical management of irAEs by allowing for earlier or prophylactic interventions to mitigate toxicities.

Although there is intense interest in identifying markers that predict the efficacy of ICIs [8, 9], pre-treatment biomarkers of ICI toxicity and irAEs have been less thoroughly investigated. Changes in IL-17, CD8 T-cell clonal expansion, eosinophil counts, and markers of neutrophil activation have been associated with specific irAEs after treatment induction, but did not predict toxicity development when tested at baseline [10–12]. Several other potential baseline risk factors for development of irAEs from ICI have been suggested, including a family history of autoimmune diseases, previous viral infections, and use of medicines with known autoimmune toxicities [13, 14], but these require further validation. More recently, in a small study, the baseline microbiome composition of melanoma patients was found to be associated with onset of immune mediated colitis following anti-CTLA-4 treatment [15]; while this finding demonstrates the potential utility of pre-treatment/baseline biomarkers of toxicity development, it does not reflect the spectrum of different irAEs associated with ICI. In light of the similarities in clinical presentation between patients experiencing irAEs from ICI therapy and those with autoimmune disorders, such as colitis, hepatitis, thyroiditis, nephritis, hypophysitis, rashes and arthralgias [16], we hypothesized that a subset of melanoma patients have a baseline (pre-treatment) autoimmune susceptibility, characterized by a repertoire of pre-existing autoantibodies against specific antigen targets, which can predict development of irAEs following ICI therapy. We tested this hypothesis using a human proteome microarray to identify toxicity-associated autoantibodies in pre-treatment sera from 75 metastatic melanoma patients who received anti-CTLA-4, anti-PD-1, or combination treatment (anti-CTLA-4 and anti-PD-1 together).

## Methods

### Study population and serum collection

Metastatic melanoma patients treated with ICI therapy at New York University (NYU) Langone Health from 2011 to 2016 were enrolled in the Interdisciplinary Melanoma Cooperative Group (IMCG) biospecimen database protocol. This protocol, approved by the NYU Institutional Review Board, prospectively enrolls patients with melanoma presenting to surgical and medical oncologists at the NYU Perlmutter Cancer Center (PCC), and banks patient biospecimens (linked to extensive, prospectively recorded clinicopathological data) for research purposes with protocol-driven follow up every 3 months [17]. Informed consent for use of clinical data and specimens was obtained from all patients at the time of enrollment.

To minimize pre-analytical variability, samples were routinely collected, processed, and stored using standardized NYU IMCG protocols. Blood was collected in Becton–Dickinson vacutainer SST venous blood collection—serum tubes (catalog #366430). For consistency and reproducibility, samples were processed < 90 min after collection by centrifugation for 10 min at 2500 rpm at room temperature. Aliquots (1 ml) of sera were stored in 1.8 ml cryovials at − 80 °C, and thawed once at the time of the proteomic array assay.

For assay validation purposes, two identical serum samples were collected from 10 immunotherapy treated patients: (i) anti-CTLA-4 (n = 3), (iii) anti-PD-1 (n = 3), and (iii) combination therapy (n = 4), to assess the reproducibility of the proteomic microarray. All sera were aliquoted into smaller volumes and stored at − 80 °C until further use, and thawed on ice prior to the assay.

Pre-treatment sera samples (n = 78) were prospectively collected from three different ICIbased cohorts of stage IV metastatic melanoma patients: (i) anti-CTLA-4 (n = 39 samples from 37 patients), (ii) anti-PD-1 (n = 28 samples from 27 patients), and (iii) anti-CTLA4/anti-PD-1 combination therapy (n = 11 samples from 11 patients). Patient-matched post-treatment samples were also collected for the anti-CTLA-4 cohort. Samples were grouped based on immunotherapy toxicity outcomes that were determined from treatment initiation to at least 6 months after the last treatment. Clinicians treating patients at the NYU PCC rigorously assessed toxicity according to objective common terminology criteria for adverse events (CTCAE) criteria. All patient medical records underwent additional review by a medical oncologist (MW) to account for differences in toxicity monitoring of patients treated on and off protocol. Toxicity was stratified into three clinically-relevant groups: no toxicity (CTCAE grade 0), mild toxicity (CTCAE grade 1–2), and severe toxicity (CTCAE grade 3–4).

### Serum antibody profiling using a human proteome microarray

To profile serum antibodies, we utilized a human proteome microarray (HuProt Human Proteome Microarray v3.1, CDI Labs, Mayaguez, PR) that contains over 19,000 unique, individually-purified full-length human proteins in duplicate, covering more than 75% of the proteome [18]. Briefly, the HuProt arrays were blocked with blocking solution (5% BSA/1×TBS-T) at room temperature for 1 h, and then probed with serum samples (diluted 1:1000) at 4 °C overnight. The arrays were then washed with 1×TBS-T for 3 times, 10 min each, and probed with Alexa-647 labeled anti-human IgG (Jackson ImmunoResearch, West Grove, PA) at room temperature for 1 h, followed by three washes of 1×TBS-T, 10 min each, and then spun to dryness prior to scanning.

### Array data pre-processing

Slides were scanned using a GenePix 4000B instrument (Molecular Dynamics, Sunnyvale, CA) and GenePix Pro (v7.2.22) software was used to measure the signal intensities for IgG binding to array features as well as any background signal present. Before pre-processing, each array was manually inspected and problematic probes were flagged. For each sample array, resulting GPR files were processed using the Bioconductor (v3.5) package PAA (v1.10.0) in R (v3.4.1).

To assess the overall quality of individual arrays, foreground and background signal intensities were plotted by array position to determine any regions containing technical artifacts. These regions were noted and compared to array plots made following all preprocessing to assess the cumulative effect of all procedures on individual arrays. The signal intensities for probes which had been manually flagged were replaced by the median signal intensity for all probes which were not flagged, and arrays were subsequently corrected for background intensities using the Bioconductor package limma (v3.32.5) with the “normexp” model and a saddle-point approximation. To determine the appropriate normalization procedure, MA plots were created for each sample array and the effects of cyclic loess, quantile, and vsn normalization visualized. Cyclic loess normalization gave the best normalization across all arrays and was applied using the normalizeArrays function in the PAA package. Finally, a combined signal intensity was generated from the duplicate probes for each antibody using the mean of the individual signal intensities and changing to log_2_ scale.

### Analysis of differential levels of serum antibodies

For each treatment type, pre-treatment samples were assigned to one of two toxicity groups (no/mild toxicity versus severe toxicity) for differential expression analysis. Student’s t test was used to determine if there was a significant difference between average signal intensities for each antibody across toxicity groups, and p values and log_2_ fold change (FC) were recorded for each antibody. The power calculations for comparing the toxicity groups for the three treatments are shown in Additional file 1: Table S1, and indicate that the studied sample sizes are adequately powered (≥ 80%) to detect antibodies with FCs at 1.15, 1.13 and 1.48 at alpha = 0.01 for the anti-CTLA-4, anti-PD-1, or combination treatments, respectively. Antibodies with p < 0.05 between toxicity groups were defined as being differentially expressed (DE), and those with p < 0.01 and |log_2_(FC)| > log_2_(1.5) were designated as belonging to a “filtered” list of DE antibodies associated with toxicity. Both DE and “filtered” antibodies were used in further analyses.

### Classification models for treatment toxicity

For each treatment type and each antibody in the “filtered” differentially expressed list, the Shannon entropy was calculated and information gain derived. Information gain describes how important a particular feature (antibody) is with regards to the model being developed. Any antibodies with corresponding information gain > 0.05 were retained as a part of a “curated” antibody feature set. While this threshold is low, it was necessary due to the relatively low number of samples available and still enables the identification of antibodies involved in toxicity prediction.

Using the “filtered” and “curated” antibody sets separately, two support vector machine (SVM) classification models were built using R package e1071 (v1.6.8) with type parameter C-classification and radial bias kernel. For each model, samples were divided into either three or fivefolds, depending on the number of samples available in each toxicity group, and cross-validation used to assess model performance. Each fold was left out for testing once, and a model trained using the remaining folds. Every model was evaluated for training and testing accuracy, sensitivity, and specificity, and for each sample the probability of being designated severe toxicity was recorded. Samples with no/mild toxicity were designated as “negative” and those with severe toxicity designated as “positive”; therefore, sensitivity describes the proportion of severe toxicity samples accurately identified while specificity describes the proportion of no/mild toxicity samples identified as such. This three or fivefold cross-validation scheme was repeated 100 times in order to mitigate the effects of overfitting due to limited sample numbers. By repeating the cross-validation procedure and reporting the average results, it is possible to ensure that reported statistics are not overestimated due to how samples are assigned to training versus testing groups.

Functional analysis of antigen targets of toxicity-associated antibodies. The HOMER (v4.9) enrichment analysis tool and functional annotations from WikiPathways (http://www.wikipathways.org/) were used to determine the potential significance of the antigen targets of antibodies that were strongly differentially expressed between no/mild and severe toxicity groups.

## Results

### Reproducibility of a proteomic microarray for serum antibody profiling

**Fig. 1:**
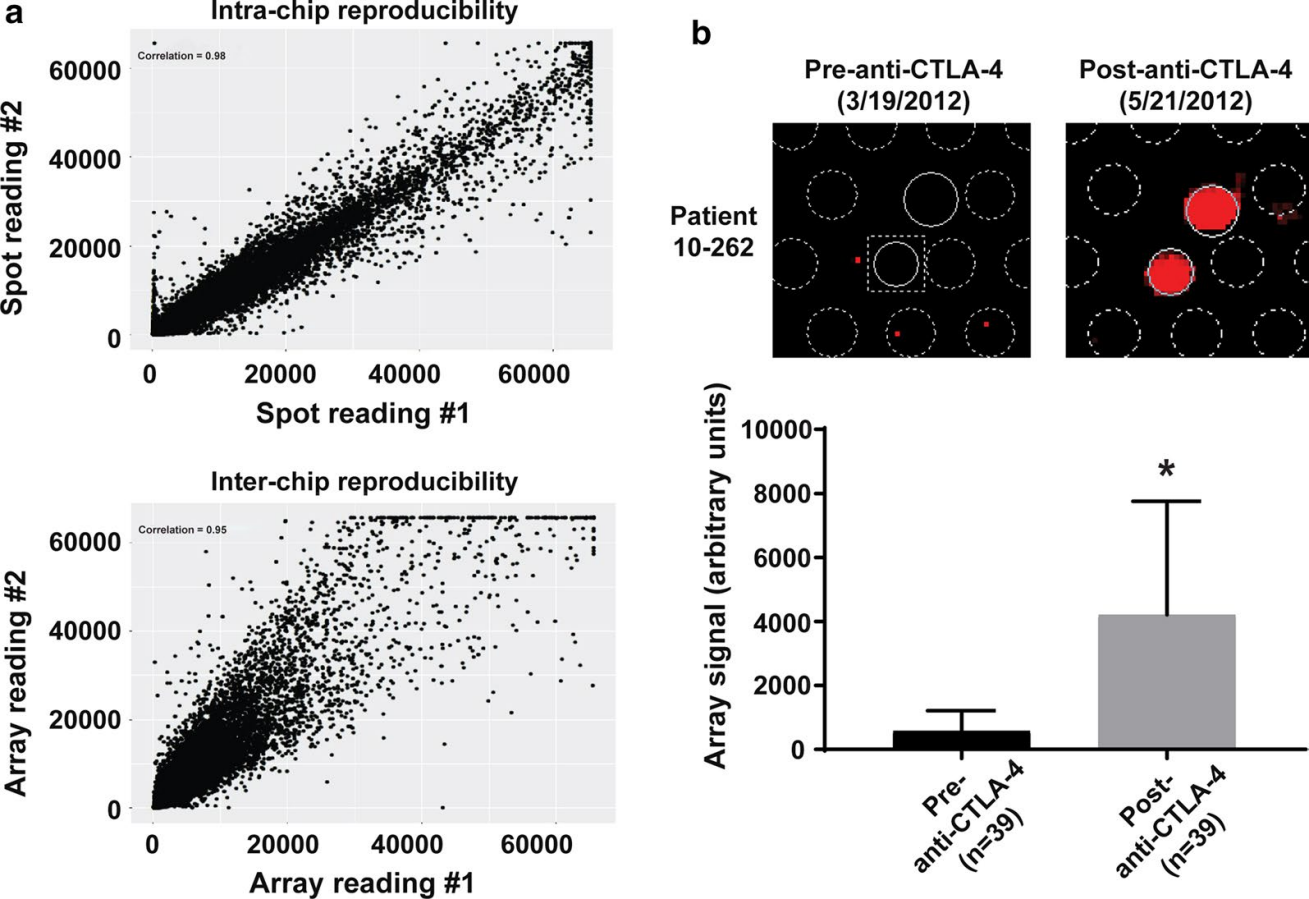
Validation of a proteomic microarray for measurement of serum antibodies. **a** Intra-chip reproducibility was assessed by comparing probe intensity readings for duplicate spots from 10 independent serum samples/chips. Linear regression analysis was used to determination the correlation between spots within chips. To assess interchip reproducibility, probe intensity readings were assessed in the same 10 serum samples across two distinct microarrays on separate occasions, and linear regression analysis was used to determine the correlation between chips. **b** Comparison of probe array signal intensities for anti-CTLA-4 antibodies from serum samples (n = 39) from melanoma patients taken before and after anti-CTLA-4 ICI treatment. Top, raw array scans of duplicate anti-CTLA-4 spots for pre- and post-anti-CTLA-4 samples from patient 10-262. Bottom, graph showing combined anti-CTLA-4 array signals (mean ± SD) for all pre- and post-treatment samples. *p < 0.0001

As assay reproducibility is critical for biomarker development, we first assessed the intra-chip and inter-chip reproducibility of a human proteome microarray (HuProt v3.1, CDI Labs) using pre-treatment sera from a cohort of 10 metastatic melanoma patients (Additional file 2: Table S2). We assessed the correlation between duplicate immunoglobulin G (IgG) spots on each chip and found a high degree of intra-chip reproducibility (r^2^ = 0.98; Fig. 1a, top). The same 10 sera samples were also assayed on two separate occasions to assess inter-chip reproducibility, which showed a strong correlation between IgG antibody readings across chips (r^2^ = 0.95; Fig. 1a, bottom). We then tested anti-CTLA-4 IgG antibody levels between matched pre- and posttreatment sera from an independent anti-CTLA-4 cohort (n = 39 samples) as an internal control, and found that anti-CTLA-4 IgG antibody levels were significantly increased in post-treatment vs. pre-treatment sera (p < 0.0001; Fig. 1b). Our analysis also showed a strong correlation (mean r^2^ = 0.89) between global IgG antibody levels from pre- and post-anti-CTLA-4 treatment sera (Additional file 3: Figure S1). Hence, the human proteome microarray allows reproducible and sensitive profiling of serum autoantibodies, making it suited to identification of differences in pre-treatment autoantibody levels in patient sera.

### Differences in baseline serum autoantibodies predict development of immunotherapy toxicity

To test our hypothesis that a specific baseline autoantibody profile can predict development of toxicity following treatment with ICI, we assessed IgG antibody levels in 78 baseline serum samples from 75 ICI-treated metastatic melanoma patients. We assayed 39 serum samples from 37 anti-CTLA-4-treated patients, 28 serum samples from 27 patients treated with anti-PD-1, and 11 samples from 11 patients treated with combined anti-CTLA-4/anti-PD-1 (Additional file 4: Table S3). The severity of immune toxicity was graded according to objective common terminology criteria for adverse events (CTCAE), following detailed review of patient medical records by a single investigator (MW), as either no toxicity (grade 0), mild toxicity (grade 1–2) or severe toxicity (grade 3–4). We also noted the location and type of immune toxicity (gastrointestinal, skin, endocrine) experienced by each patient. Comparing patients treated with anti-CTLA-4, anti-PD-1, or combined anti-CTLA-4/anti-PD-1, there was no statistically significant difference in gender, age at treatment initiation, pre-treatment lactate dehydrogenase (LDH) levels, or pre-treatment Eastern Cooperative Oncology Group Performance Status (ECOG PS; [19]) (Table 1). Furthermore, we did not observe significant differences in the severity or location of toxicity between treatment groups. Compared to anti-CTLA-4 or anti-PD-1 monotherapy patients, the combination treatment cohort showed significantly better response to therapy (p = 0.01) but also significantly more treatment termination (p = 0.006), which is consistent with clinical trials demonstrating the greater efficacy and increased toxicity with combined ICI [2].

**Table 1:** Baseline patient characteristics

		Anti-CTLA-4	Anti-PD-1	Combination	Fisher’s test
		(n = 37)	(n = 27)	(n = 11)	p value
		No. (%)	No. (%)	No. (%)	
Gender	Female	12 (32)	11 (41)	4 (36)	0.848
	Male	25 (68)	16 (59)	7 (64)	
Age at treatment initiation	Mean (SD)	66.2 (13)	69.9 (14)	59.9 (13)	1
	Median	67.4	71	61.1	
ECOG PS (pretreatment)	0	28 (76)	19 (70)	7 (64)	0.736
	> 1	9 (24)	8 (30)	4 (36)	
LDH (pretreatment)	Normal	31 (91)	19 (70)	9 (82)	0.114
	Elevated	0 (9)	8 (30)	2 (18)	
	Unknown	3	0	0	
Response to treatment	POD	22 (59)	10 (37)	4 (36)	0.01
	SD	10 (27)	5 (19)	1 (9)	
	PR	5 (14)	8 (30)	2 (18)	
	CR	0	2 (7)	4 (36)	
	UNC	0	2 (7)	0	
Toxicity	None	8 (22)	4 (15)	0	
	Mild	20 (54)	15 (55)	4 (36)	0.16
	Severe	9 (24)	8 (30)	7 (64)	
GI toxicity	Mild	9 (23.1)	12 (42.9)	3 (27.3)	0.08
	Severe	6 (15.4)	3 (10.7)	6 (54.5)	
Skin toxicity	Mild	15 (38.5)	17 (60.7)	5 (45.5)	0.43
	Severe	0	1 (3.6)	1 (9.1)	
Endocrine toxicity	Mild	5 (12.8)	11 (39.2)	4 (36.4)	0.71
	Severe	0	1 (3.6)	1 (9.1)	
Required treatment termination	Yes	4 (11)	3 (11)	6 (54)	0.006
	No	33 (89)	24 (89)	5 (46)	

Summary of clinical features from 75 melanoma patients treated with anti-CTLA-4 (n = 37), anti-PD-1 (n = 27), or anti-CTLA-4 and anti-PD-1 (n = 11). *LDH* lactate dehydrogenase, *POD* progression of disease, *SD* stable disease, *PR* partial response, *CR* complete response, *UNC* unclassified. Fisher’s exact test was used to examine the significance of the association between patient characteristics and treatment type. Two anti-CTLA-4 patients were sampled twice (11-311, in 2011 and 2013; 12-071, in 2012 and 2013), and one anti-PD-1 patient was sampled twice (13-185, in 2015 and 2016)

**Fig. 2:**
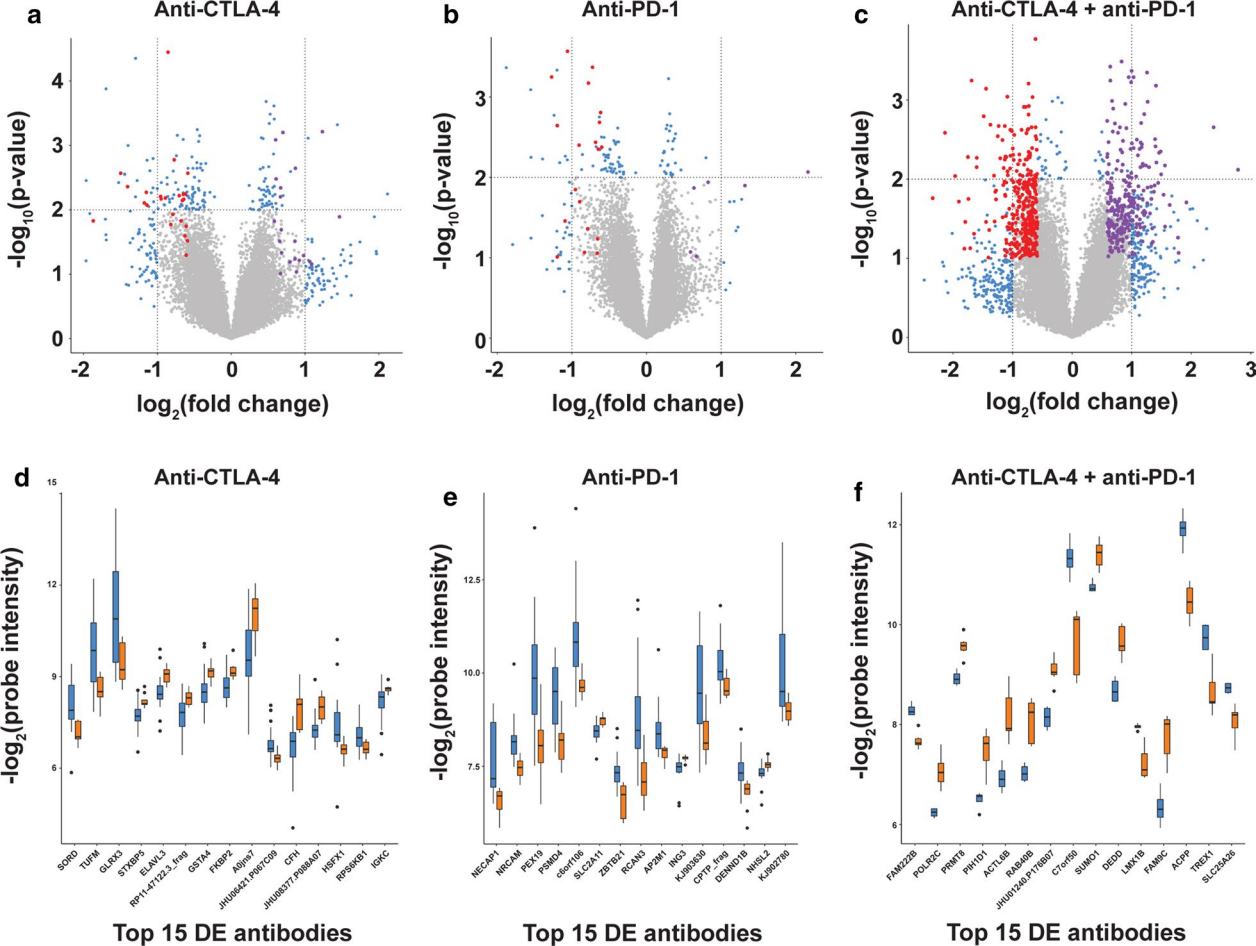
Antibodies from baseline sera of melanoma patients are associated with ICI toxicity. **a** Volcano plot of differential antibody levels from baseline sera comparing none/mild vs. severe toxicity for anti-CTLA-4-treated patients (n = 37). Filtered antibodies are highlighted in blue, and curated antibodies are indicated in red (downregulated with severe toxicity) or purple (upregulated with severe toxicity). **b** As for **a**, but comparing no/mild vs. severe toxicity for anti-PD-1-treated patients (n = 27). **c** As for **a**, but comparing mild vs. severe toxicity for anti-CTLA-4 and anti-PD-1 combination treated patients (n = 11). **d** Boxplots showing probe intensities for the 15 most differentially expressed antibodies (DE; based on p values) between sera from antiCTLA-4 patients (n = 37) with no/mild toxicity (blue) vs. those with severe toxicity (orange). Data represent median probe intensities ± sd. **e** As for **d**, but for samples comparing no/mild vs. severe toxicity for anti-PD-1-treated patients (n = 27). **f** As for **d**, but for samples comparing mild vs. severe toxicity for combination anti-CTLA-4 and anti-PD-1-treated patients (n = 11)

To identify pre-immunotherapy toxicity-associated autoantibodies, we compared IgG autoantibody profiles between anti-CTLA-4- or anti-PD-1-treated patients who experienced no or mild vs. severe toxicity. For pre-treatment samples from the combined anti-CTLA-4 and anti-PD-1 treatment group, we compared IgG antibodies between mild and severe toxicity samples, as all patients developed some degree of immune-related toxicity with this regimen. We observed toxicity-associated differences in IgG antibody levels for each ICI treatment (Fig. 2a–c), and set two thresholds for differential antibody expression for each comparison based on power calculations derived from experimental data. Differentially expressed (DE) antibodies were defined as those with p < 0.05 between no/mild and severe toxicity (Fig. 2d–f). We identified 914 DE antibodies associated with severe toxicity in the anti-CTLA-4 cohort, 723 DE antibodies associated with severe toxicity in the anti-PD-1 cohort, and 1161 DE antibodies associated with severe toxicity in the combination treatment cohort (Additional file 5: Table S4 and Additional file 6: Table S5). Interestingly, we observed a minimal degree of overlap in toxicity-associated IgG antibodies (DE) between monotherapy groups (antiCTLA-4 or anti-PD-1) and the combination therapy (anti-CTLA-4 + anti-PD-1) group. For example, there were only 99 IgG antibodies in common between 849 unique anti-CTLA4 toxicity-associated IgG antibodies and 1071 unique anti-CTLA-4 and anti-PD-1 toxicity-associated antibodies. Similarly, there were only 54 IgG antibodies in common between 683 unique anti-PD-1 toxicity-associated IgG antibodies and 1071 unique anti CTLA-4 and anti-PD-1 toxicity-associated antibodies (data not shown). This suggests that discrete, treatment type-specific sets of antibodies are associated with ICI toxicity.

**Fig. 3:**
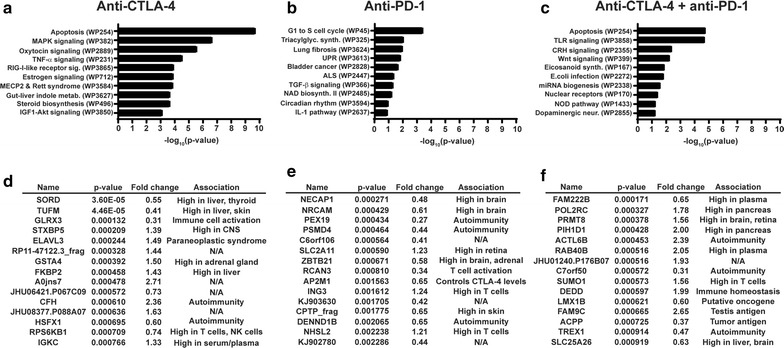
Functional significance of toxicity-associated antibodies. **a** Functional pathway enrichment (WikiPathways) of protein targets from the filtered set of toxicity-associated antibodies from anti-CTLA-4-treated patients. **b** As for **a**, but for anti-PD-1-treated patients. **c** As for **a**, but for combination-treated patients. **d** Summary of immune toxicity associations for protein targets of top 15 DE termination-associated antibodies from anti-CTLA-4-treated patients. **e** As for **d**, but for anti-PD-1-treated patients. **f** As for **d**, but for combination-treated patients

To gain insight into potential causative roles for toxicity-associated antibodies in development of irAEs, we performed pathway analysis on the protein antigen targets identified for each treatment group. We elected to focus our analysis on the filtered sets of toxicity-associated antibodies for each treatment type, as defined above. Our results revealed significant enrichment of proteins in pathways that have been previously associated with immunity/autoimmunity (Additional file 7: Table S6), including “apoptosis”, “TNF-α signaling”, “lung fibrosis”, “IL-1 pathway”, “toll-like receptor (TLR) signaling”, “*E. coli* infection”, and “microRNA biogenesis” (Fig. 3a–c). A literature analysis for the fifteen most DE toxicity-associated antibodies for each treatment group (Fig. 2d–f) revealed that their protein targets were highly expressed in tissues that are commonly affected in patients experiencing irAEs, including liver and skin, and have been implicated in the regulation of immune cell activity and in autoimmune disorders (Fig. 3d–f, and Additional file 8: Table S7). Together, our findings suggest that a subset of toxicity-associated antibodies could not only highlight patients at risk of irAEs from immunotherapy, but might also play a causative role in the development of immune toxicity.

**Fig. 4:**
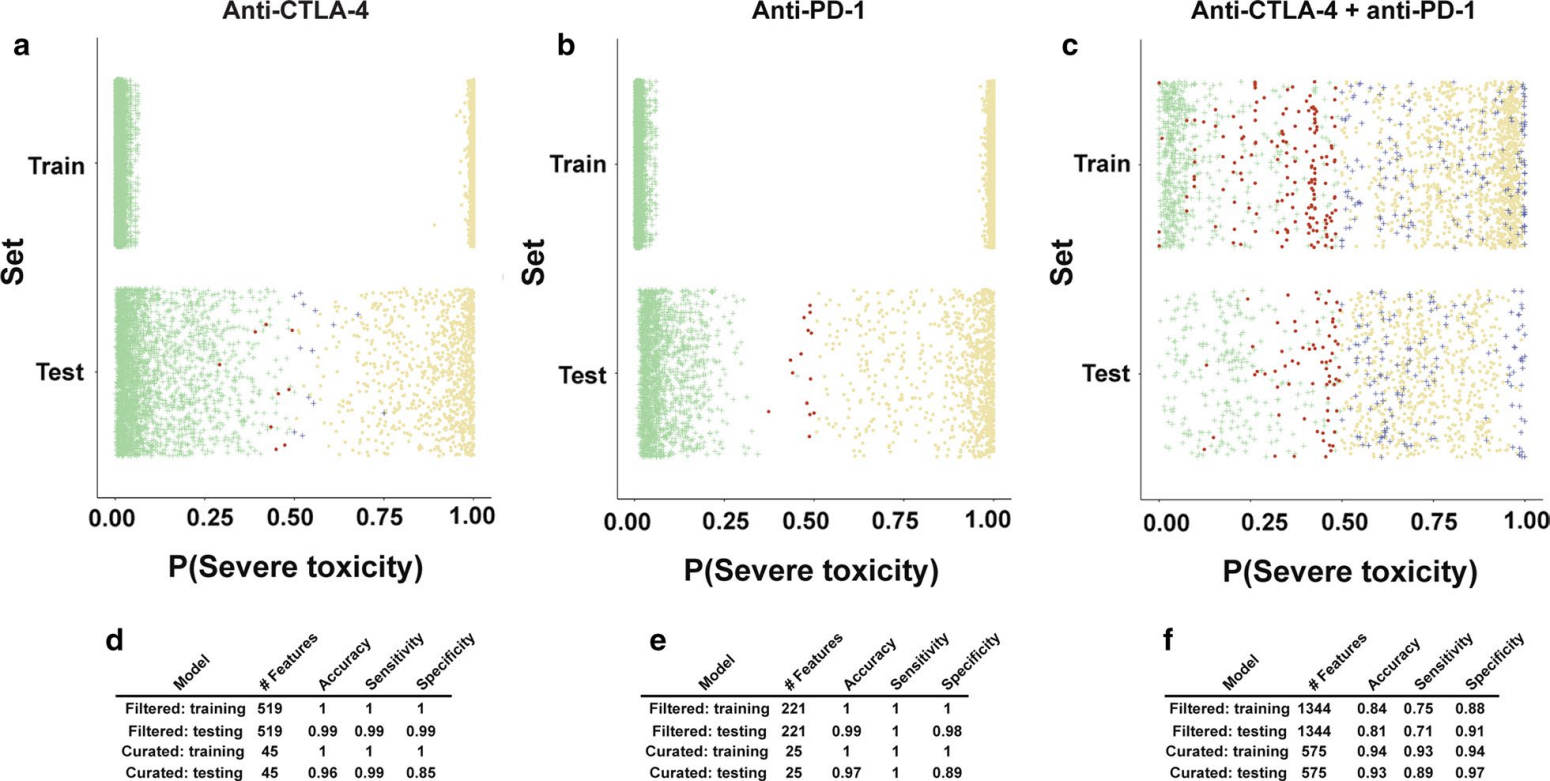
Development of classification models to predict immunotherapy toxicity using antibodies from pre-treatment melanoma patient sera. **a** Scatterplot showing distribution of decision values from support vector machine (SVM) classifier models based on “filtered” antibody (feature) lists for prediction of severe toxicity. Data summarizes training and testing results from 100 repetitions of fivefold cross validation for pre-anti-CTLA-4 samples. Gold circles represent true positives (severe toxicity sample called as severe toxicity) and green crosses represent true negatives (no/mild toxicity sample called as no/mild toxicity). Red circles represent false negatives (severe toxicity sample called as no/mild toxicity) and blue crosses represent false positives (no/mild toxicity called as severe toxicity). **b** As for **a**, but summarizing 100 repetitions of fivefold cross validation for anti-PD-1 samples. **c** As for **a**, but summarizing 100 repetitions of threefold cross validation for anti-CTLA-4 and anti-PD-1 combination samples. **d** Summary of accuracy, sensitivity, and specificity cross validation statistics based on SVM models for prediction of toxicity in anti-CTLA-4 samples (no/mild toxicity, n = 30; severe, n = 9). **e** As for **d**, but for anti-PD-1 samples (no/mild toxicity, n = 19; severe, n = 9). **f** As for **d**, but for combined anti-CTLA-4 and anti-PD-1 samples (mild toxicity, n = 4; severe, n = 7)

To develop a tool to predict toxicity development in melanoma patients treated with ICI, we derived support vector machine (SVM) classification models to classify patients according to their risk of developing severe immunotherapy-related toxicity based on the levels of specific antibodies (features) in baseline sera. We performed SVM model training and testing for each treatment group using “filtered’ and “curated” feature lists (as defined above). For each model, we used threefold (combination therapy) or fivefold (monotherapy) cross-validation and repeated the scheme 100 times to mitigate the impact of overfitting (Fig. 4a–c). “Filtered” antibody sets predicted severe toxicity development with excellent (> 0.98) accuracy, sensitivity, and specificity for antiCTLA-4 (Fig. 4d) and anti-PD-1 (Fig. 4e) monotherapy groups, and with good (> 0.71) accuracy, sensitivity, and specificity for the smaller group of combined anti CTLA-4 and anti-PD-1 patients (Fig. 4f). The prediction models we derived using the smaller “curated” antibody sets (n = 45 for anti-CTLA-4, n = 25 for anti-PD-1, n = 575 for combination treatment) showed very good (> 0.85) accuracy, sensitivity, and specificity for all three treatment groups (Fig. 4d–f). These results suggest that baseline antibody signatures should be evaluated further for their clinical utility as biomarkers to predict toxicity from immunotherapy.

## Discussion

Immune-related toxicities are a significant barrier limiting the utility of ICI in melanoma treatment, particularly when given in combination [20]. At this time, there is no predictive biomarker to identify patients who are likely to develop severe irAEs that can necessitate systemic immunosuppression or treatment termination. We hypothesized that a subset of metastatic melanoma patients possesses a sub-clinical autoimmune phenotype, characterized by a specific serum autoantibody profile, which predisposes them to develop severe irAEs following ICI therapy, in part due to enhanced recognition of self-antigens by T-cells. We used an unbiased proteomic microarray approach to compare global antibody levels in pre-treatment sera from melanoma patients treated with anti-CTLA-4, anti-PD-1, or the combination, and identified sets of toxicity-associated antibodies for each of the three treatment cohorts. Interestingly, the toxicity-associated antibody signatures were treatment-specific, with very little overlap across therapy groups, a finding that might be explained by the distinct cellular mechanisms of action for these treatments [21]. We found that the antigen targets for toxicity associated autoantibodies were significantly enriched for proteins that are highly expressed in organs affected by irAEs, and/or involved in cellular pathways that have been associated with immune pathology, suggesting a potential causative role for specific autoantibodies in development of irAEs. Finally, we generated SVM classifier models to identify sets of features (antibodies) that could be used to predict toxicity from baseline sera with excellent accuracy, sensitivity and specificity, demonstrating the potential utility of this approach to develop biomarker assays to guide the clinical management of melanoma patients treated with ICI.

By reinvigorating the immune system, immunomodulatory antibodies (anti-CTLA-4, anti PD-1) can promote anti-tumor immunity but also the development of irAEs. The precise mechanisms underlying irAEs induced by ICI are still unclear. Gastrointestinal (GI) irAEs have been associated with increased levels of neutrophil activation markers CD177 and CEACAM1, which are correlated with neutrophilic inflammation [10]. Additionally, it has been suggested that high baseline serum levels of IL-17, a cytokine that activates neutrophils, are associated with development of colitis following antiCTLA-4 treatment [12]. A recent report also suggested that hypophysitis following antiCTLA-4 treatment might be associated with development of antibodies, which were negative at baseline, against thyrotropin-, follicle-stimulating hormone-, and corticotropin-secreting pituitary gland cells [22]. While these pituitary-specific antibodies might mediate this irAE, they cannot be utilized as a predictive biomarker of treatment-induced pituitary toxicity as they are not detectable in pre-treatment sera. Other studies have failed to identify baseline serum antibodies associated with development of irAEs in immunotherapy-treated patients, although these focused solely on antibodies previously implicated in autoimmune diseases, such as anti-nuclear [23] or anti-thyroid [24] antibodies. ANAs, targeting both nuclear proteins and nucleic acid derivatives, comprise a large proportion of autoimmune disease-associated antibodies and have the most-recognized diagnostic and/or prognostic value for autoimmune diseases such as systemic lupus erythematosus (SLE) [25].

Our analyses identified significant enrichment of the protein targets of toxicity associated antibodies among functional pathways that have been associated with autoimmunity, such as TNF-α signaling [26], lung fibrosis [27], IL-1 [28] and TLR [29] signaling, and *E. coli* infection [30]. Interestingly, our results also showed that the most differentially-expressed antibodies between no/mild and severe toxicity groups for each therapy group target protein antigens that are highly expressed in tissues affected by irAEs, including liver, skin, thyroid, pancreas, and adrenal gland [31]. In this regard, it is interesting to speculate that specific baseline antibodies could predict the development of severe site-specific toxicities that are more clinically-significant; for example, severe hepatotoxicity versus severe skin toxicity, although this would require further testing and validation in a larger sample size. Many of the putative protein targets of the antibodies most significantly-associated with severe toxicity have been implicated in immune function or in autoimmune disorders. For example, autoantibodies against the complement factor H (CFH) protein have been associated with autoimmune diseases such as hemolytic uremic syndrome, membranoproliferative glomerulonephritis, or age-related macular degeneration [32], and levels of anti-CFH antibodies were elevated in our study in patients who developed severe toxicity from anti-CTLA-4 treatment. In this regard, it would be informative to compare serum antibody profiles from patients who developed severe toxicity from ICI therapy to those with autoimmune disease states, such as SLE or inflammatory bowel disease. While the precise roles of toxicity-associated antibodies we identified in mediating irAEs are yet to be established, their potential biological significance supports the concept that a subset of antibodies could promote the development of irAEs in patients treated with ICI. In view of the underlying similarities between the clinical manifestation of autoimmune disorders and irAEs induced by ICI, our data support a model in which some ICI-treated melanoma patients possess an underlying, subclinical autoimmune phenotype, which renders them susceptible to severe irAE development and is characterized by a specific baseline serum antibody profile. This autoimmune phenotype is likely to result from a combination of host- (germline), environment-, and tumor-specific factors. We previously reported the association between germline genetic variants in immune pathways and melanoma prognosis [33, 34]. Thus it is possible that an inherent genetic repertoire may generate a propensity for host immunity and as such might impact production of antibodies, including those that target putative “self” antigens. Parallel assessment of the inherited genome (in the context of autoimmunity risk), the proteome, and immune profile (in tumor and circulation) is required to assess the causative biological role of “baseline” host immunity in development of irAEs in patients treated with ICI.

We acknowledge the limitations to our study. First, the findings should be replicated with a larger, independent cohort. Ideally, this clinical validation would involve a retrospective study of patient sera from a large clinical trial, where toxicity grading, treatment, and baseline patient characteristics are rigorously graded and controlled. Clinical validation would also require prospective testing before serum antibody biomarkers could have utility as predictors of ICI toxicity development. Second, our study does not address the contribution of host genetic factors as determinants or predictors of immune toxicity. Third, although our data suggest that baseline antibody levels can predict irAEs from ICI, the proteomic array platform we used cannot assess levels of anti-nucleotide or anti-lipid autoantibodies, which have been shown to have diagnostic and prognostic value in various autoimmune diseases. Nevertheless, the association between levels of specific antibodies and immunotherapy toxicity development in our study, together with the SVM classification models we derived, suggests that predictive antibody panels can be used to differentiate between melanoma patients based on their likely susceptibility to severe irAEs from ICI. As there is no existing biomarker for immunotherapy toxicity in clinical use, at this time there is no standard-of-care benchmark with which we can compare the predictive power of our sets of antibodies. Ultimately, validation of these predictive biomarkers could enable clinicians to optimize the risk–benefit assessment for individual patients to maximize therapeutic benefit while minimizing possible severe toxicities from ICI. Patients who are likely to develop severe irAEs could undergo treatment modification, closer clinical monitoring, earlier prophylactic use of therapies (e.g. corticosteroids, anti-TNFα), or could avoid combination ICI to mitigate severe irAEs. As checkpoint inhibitors are increasingly used in a range of other cancer types, including bladder, lung, head and neck, renal, and microsatellite instability (MSI) high GI cancers [35, 36], the absolute number of patients exposed to ICI toxicity will increase, and it will be interesting to determine whether there is a common signature of toxicity associated antibodies for a given ICI regimen across different cancer types. Furthermore, as ICI enter the adjuvant treatment setting for melanoma [6, 7] there is a crucial need to predict and limit the exposure of patients to severe toxicity. In conclusion, our results provide an important foundation to develop robust pre-treatment biomarkers to predict irAE development in metastatic melanoma patients treated with ICI, which would ultimately improve personalized immunotherapy and management of irAEs.

## Conclusions

Currently, there is no predictive biomarker to identify patients who are likely to develop severe irAEs that can necessitate systemic immunosuppression or treatment termination. In this study we showed that a subset of metastatic melanoma patients, display a specific serum autoantibody profile, which predisposes them to developing severe irAEs following ICI therapy. We used an unbiased proteomic microarray approach to compare global antibody levels in pre-treatment sera from melanoma patients treated with anti-CTLA-4, anti-PD-1, or the combination, and identified sets of toxicity-associated antibodies for each of the three treatment cohorts. We found that the antigen targets for toxicity associated autoantibodies were significantly enriched for proteins that are highly expressed in organs affected by irAEs, and/or involved in cellular pathways that have been associated with immune pathology. Finally, we generated SVM classifier models to identify sets of features (antibodies) that could be used to predict toxicity from baseline sera with excellent accuracy, sensitivity and specificity. As the use of immunotherapies is expanded to other cancers and the adjuvant setting there is a growing need for predictive toxicity biomarkers that could help clinicians to determine the risk–benefit ratio for individual patients to maximize therapeutic benefit while minimizing severe toxicities.

## Additional files

**Additional file 1: Table S1.** Detectable fold-changes (FC) at 80% power between toxicity groups for the three treatments. Power calculations for comparison of antibody levels between no/mild versus severe toxicity for the three ICI treatments.

**Additional file 2: Table S2.** Patient characteristics for reproducibility cohort (n = 10). Summary of clinical features from independent group of 10 melanoma patients treated with anti-CTLA-4 (n = 3), anti-PD-1 (n = 3), or combined anti-CTLA-4/anti-PD-1 (n = 4), and from whom serum samples were used to assess assay reproducibility. LDH, lactate dehydrogenase; POD, progression of disease; SD, stable disease; PR, partial response; CR, complete response; UNC, unclassified.

**Additional file 3: Figure S1.** Pre- vs. post-anti-CTLA-4 treatment reproducibility (n = 39). (A) Correlation plot of global antibody profiles (array probe intensities) for pre- and postCTLA-4 treatment samples from patient 09-035. (B) Summary of correlation (r^2^) values for antibody profiles, including mean and standard deviation, between pre- and postanti-CTLA-4 treatment samples (n = 39 pairs).

**Additional file 4: Table S3.** Sample details. Severity and site of toxicity (gastrointestinal (GI), endocrine, skin and/or other) and treatment termination status for baseline sera samples from anti-CTLA-4 (n = 39), anti-PD-1 (n = 28), and combination (n = 11) melanoma patients. Two anti-CTLA-4 patients were sampled twice (11-311, in 2011 and 2013; and 12-071, in 2012 and 2013), and one anti-PD-1 patient was sampled twice (13-185, in 2015 and 2016).

**Additional file 5: Table S4.** Summary of toxicity- and termination-associated antibodies. Numbers of differentially expressed (DE), strongly differentially expressed (strong DE), filtered and curated antibodies are shown for comparisons of none/mild vs. severe toxicity, across three different treatment groups (anti-CTLA-4, anti-PD-1, and combination).

**Additional file 6: Table S5.** Toxicity- and termination-associated antibodies. Lists of differentially expressed, strongly differentially expressed, filtered and curated antibodies associated with severe toxicity for anti-CTLA-4, anti-PD-1, or the combination.

**Additional file 7: Table S6.** Pathway analysis of protein targets of toxicity-associated antibodies. Lists of functional pathways (derived from WikiPathways; http://www.wikipathways.org/) enriched for protein targets of filtered toxicity-associated antibodies from anti-CTLA-4, anti-PD-1, or combination treatment groups.

**Additional file 8: Table S7.** Functions of protein targets of treatment termination-associated antibodies. Functional analysis of protein targets for top 15 DE toxicity-associated antibodies for each of the anti-CTLA-4, anti-PD-1, and combination treatment groups. Associations of each antibody target with immune toxicity are given, based on literature findings.

## Abbreviations

ICI: immune checkpoint inhibitors
CTLA-4: cytotoxic T lymphocyte associated antigen 4
PD-1: programmed cell death protein 1
irAEs: immune-related adverse events
NYU: New York University
IMCG: Interdisciplinary Melanoma Cooperative Group
PCC: Perlmutter Cancer Center
CTCAE: common terminology criteria for adverse events
FC: fold change
DE: differentially expressed
SVM: support vector machine
IgG: immunoglobulin G
LDH: lactate dehydrogenase
ECOG: Eastern Cooperative Oncology Group
PS: performance Status
TLR: toll-like receptor
GI: gastrointestinal
ANA: anti-nuclear antibody
SLE: systemic lupus erythematosus
CFH: complement factor H
MSI: microsatellite instability
