# Supplementary material for: Baseline antibody profiles predict toxicity in melanoma patients treated with immune checkpoint inhibitors
Source: J Transl Med. 2018 Apr 2;16:82. doi: 10.1186/s12967-018-1452-4 (PMC5880088; doi:10.1186/s12967-018-1452-4)
Supplement: Supplementary file 1 — Additional file 1: Table S1. Detectable fold-changes (FC) at 80% power between toxicity groups for the three treatments. Power calculations for comparison of antibody levels between no/mild versus severe toxicity for the three ICI treatments. [file 12967_2018_1452_MOESM1_ESM.docx]

**Table S1**

| **Treatment Group** | **Alpha** | **N1** | **N2** | **SD of the difference in log-exp** | **COV*** | **Detectable FC*** |
| --- | --- | --- | --- | --- | --- | --- |
| anti-CTLA-4 | 0.01 | 9 | 30 | 0.1291 | 13% | 1.152 |
|  | 0.05 | 9 | 30 | 0.1291 | 13% | 1.193 |
| anti-PD-1 | 0.01 | 9 | 19 | 0.1024 | 10% | 1.125 |
|  | 0.05 | 9 | 19 | 0.1024 | 10% | 1.159 |
| combination treatment | 0.01 | 4 | 7 | 0.1507 | 15% | 1.483 |
|  | 0.05 | 4 | 7 | 0.1507 | 15% | 1.342 |
| *: calcuated using the relationship between the means and variances of Y and X = log(Y): COV(Y) = √[Exp{σ(X)²} - 1]. | | | | | | |
| *: PASS 14 Power Analysis and Sample Size Software (2015). NCSS, LLC. Kaysville, Utah, USA, ncss.com/software/pass. | | | | | | |
