# Supplementary material for: Baseline antibody profiles predict toxicity in melanoma patients treated with immune checkpoint inhibitors
Source: J Transl Med. 2018 Apr 2;16:82. doi: 10.1186/s12967-018-1452-4 (PMC5880088; doi:10.1186/s12967-018-1452-4)
Supplement: Supplementary file 2 — Additional file 2: Table S2. Patient characteristics for reproducibility cohort (n = 10). Summary of clinical features from independent group of 10 melanoma patients treated with anti-CTLA-4 (n = 3), anti-PD-1 (n = 3), or combined anti-CTLA-4/anti-PD-1 (n = 4), and from whom serum samples were used to assess assay reproducibility. LDH, lactate dehydrogenase; POD, progression of disease; SD, stable disease; PR, partial response; CR, complete response; UNC, unclassified. [file 12967_2018_1452_MOESM2_ESM.docx]

**Table S2**

|  | | **anti-CTLA-4** | **anti-PD-1** | **combination** |
| --- | --- | --- | --- | --- |
|  |  | **(n=3)** | **(n=3)** | **(n=4)** |
|  |  | **No. (%)** | **No. (%)** | **No. (%)** |
| **Gender** | Female | 2 (67) | 1 (33) | 3 (75) |
|  | Male | 1 (33) | 2 (67) | 1 (25) |
| **Age at Treatment Initiation** | Mean (SD) | 55.5 (5.58) | 72.65 (17.4) | 67.1 (4.37) |
|  | Median | 56 | 82.2 | 66.9 |
| **ECOG PS (pre-treatment)** | 0 | 3 (100) | 3 (100) | 3 (75) |
|  | >1 | 0 | 0 | 1 (25) |
| **LDH (pre-treatment)** | Normal | 3 | 0 | 4 (100) |
|  | Elevated | 0 | 3 (100) | 0 |
|  | Unknown | 0 | 0 | 0 |
| **Response to treatment** | POD | 0 | 0 | 0 |
|  | SD | 2 (67) | 2 (67) | 0 |
|  | PR | 1 (33) | 1 (33) | 3 (75) |
|  | CR | 0 | 0 | 1 (25) |
|  | UNC | 0 | 0 | 0 |
| **Toxicity** | None | 0 | 0 | 0 |
|  | Mild | 2 (67) | 1 (33) | 1 (25) |
|  | Severe | 1 (33) | 2 (67) | 3 (75) |
| **GI Toxicity** | Mild | 1 (33) | 2 (67) | 1 (25) |
|  | Severe | 2 (67) | 1 (33) | 2 (50) |
| **Skin Toxicity** | Mild | 3 (100) | 1 (33) | 3 (75) |
|  | Severe | 0 | 0.0 | 0.0 |
| **Endocrine Toxicity** | Mild | 1 (33) | 1 (33) | 1 (25) |
|  | Severe | 0 | 0.0 | 1 (25) |
| **Required Treatment Termination** | Yes | 0 | 1 (33) | 0 |
|  | No | 3 (100) | 2 (67) | 4 (100) |
