# Supplementary material for: Baseline antibody profiles predict toxicity in melanoma patients treated with immune checkpoint inhibitors
Source: J Transl Med. 2018 Apr 2;16:82. doi: 10.1186/s12967-018-1452-4 (PMC5880088; doi:10.1186/s12967-018-1452-4)
Supplement: Supplementary file 3 — Additional file 3: Figure S1. Pre- vs. post-anti-CTLA-4 treatment reproducibility (n = 39). (A) Correlation plot of global antibody profiles (array probe intensities) for pre- and postCTLA-4 treatment samples from patient 09-035. (B) Summary of correlation (r2) values for antibody profiles, including mean and standard deviation, between pre- and postanti-CTLA-4 treatment samples (n = 39 pairs). [file 12967_2018_1452_MOESM3_ESM.pdf]

Supplementary Figure S1

A

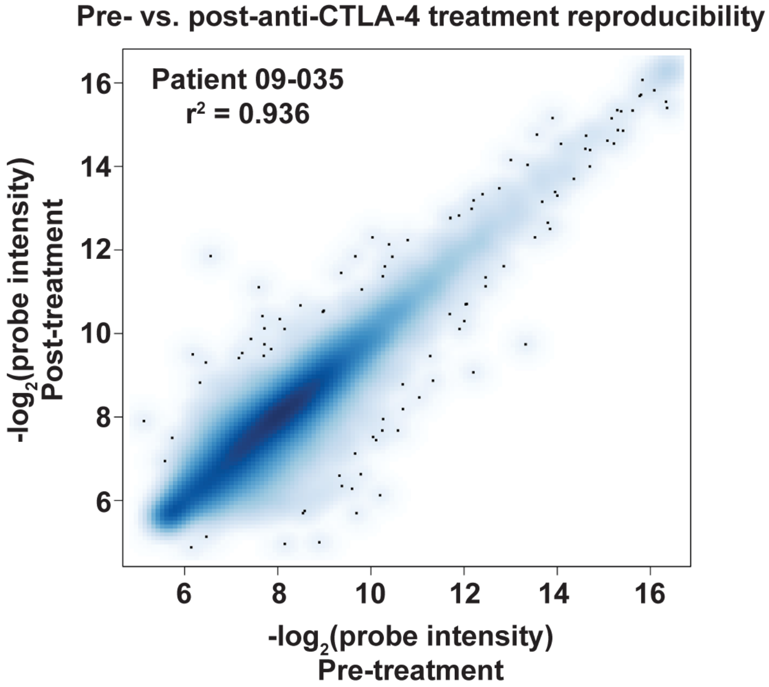

B

Patient	Correlation (r^2)
08-027	0.7683
09-035	0.936
09-213	0.9433
10-070	0.9298
10-248	0.9407
10-262	0.8467
11-018	0.8601
11-022	0.8601
11-070	0.8887
11-073	0.9598
11-086	0.9446
11-105	0.916
11-125	0.844
11-139	0.9273
11-175	0.9572
11-177	0.9508
11-246	0.9582
11-274	0.9652
11-306	0.7644
11-311 (2011)	0.7357
11-311 (2013)	0.9469

Patient	Correlation (r^2)
12-019	0.9306
12-071 (2012)	0.961
12-071 (2013)	0.962
12-074	0.9406
12-109	0.9409
12-114	0.7787
12-170	0.9243
12-185	0.9058
12-210	0.8208
13-045	0.9612
13-054	0.9423
13-019	0.7381
13-153	0.9475
13-162	0.9554
13-215	0.5625
13-219	0.9255
14-044	0.891
15-393	0.9525
Mean	0.8945
SD	0.0869
