# Supplementary material for: Baseline antibody profiles predict toxicity in melanoma patients treated with immune checkpoint inhibitors
Source: J Transl Med. 2018 Apr 2;16:82. doi: 10.1186/s12967-018-1452-4 (PMC5880088; doi:10.1186/s12967-018-1452-4)
Supplement: Supplementary file 5 — Additional file 5: Table S4. Summary of toxicity- and termination-associated antibodies. Numbers of differentially expressed (DE), strongly differentially expressed (strong DE), filtered and curated antibodies are shown for comparisons of none/mild vs. severe toxicity, across three different treatment groups (anti-CTLA-4, anti-PD-1, and combination). [file 12967_2018_1452_MOESM5_ESM.docx]

**Table S4**

| **Comparison** | **No. DE^1^** | **No. Filtered^2^** | **No. Curated^3^** |
| --- | --- | --- | --- |
| Anti-CTLA-4 –None/Mild vs. Severe | 914 | 519 | 45 |
| Anti-PD-1 –None/Mild vs. Severe | 723 | 221 | 25 |
| Anti-CTLA-4 + anti-PD-1 – Mild vs. Severe | 1161 | 1344 | 575 |

^1^ p-val < 0.05

^2^ p-val < 0.01 and |log_2_ (FC)| > log_2_ (1.5)

^3^ Selected by information gain
